# Gut-central nervous system axis is a target for nutritional therapies

**DOI:** 10.1186/1475-2891-11-22

**Published:** 2012-04-10

**Authors:** Gustavo D Pimentel, Thayana O Micheletti, Fernanda Pace, José C Rosa, Ronaldo VT Santos, Fabio S Lira

**Affiliations:** 1 Department of Internal Medicine, Faculty of Medical Sciences, State University of Campinas (UNICAMP), Campinas, SP, Brazil; 2 Faculty of Applied Science, State University of Campinas (UNICAMP), Limeira, SP, Brazil; 3 Department of Physiology and Biophysics, Institute of Biomedical Sciences I, University of São Paulo (USP), São Paulo, SP, Brazil; 4 Department of Psychobiology, Federal University of São Paulo (UNIFESP), São Paulo, SP, Brazil; 5 Laboratory of Exercise Biochemistry and Physiology, Health Sciences Unit, University of Southern Santa Catarina (UNESC), Criciúma, SC, Brazil; 6 José Caetano de Lima, 682. Bairro Junqueira, Lins MA: 16403-162, SP, Brazil

**Keywords:** Gut, Central nervous system, Nutrition, Diet, Appetite, Inflammatory disease

## Abstract

Historically, in the 1950s, the chemist Linus Pauling established a relationship between decreased longevity and obesity. At this time, with the advent of studies involving the mechanisms that modulate appetite control, some researchers observed that the hypothalamus is the "appetite centre" and that peripheral tissues have important roles in the modulation of gut inflammatory processes and levels of hormones that control food intake. Likewise, the advances of physiological and molecular mechanisms for patients with obesity, type 2 diabetes mellitus, inflammatory bowel diseases, bariatric surgery and anorexia-associated diseases has been greatly appreciated by nutritionists. Therefore, this review highlights the relationship between the gut-central nervous system axis and targets for nutritional therapies.

## Introduction

The energy balance is determined by the relationship between the acquisition and expenditure of energy. This perfect interaction occurs among physiological signals in peripheral organs and the central nervous system (CNS). Apart from the obvious digestive and absorptive functions of the gastrointestinal tract, gut and adipose tissue hormones play an important role in controlling the energy balance, particularly via the regulation of food intake in both the short- and long-term, respectively. Therefore, the enteric nervous system (ENS), gut hormones, and nutrients act in the control process at the beginning and termination of meals [1,2].

The CNS-gut axis is controlled by the ENS and its importance in the health and disease has been recognised by several studies [3,4]. According to health professionals, advances in the physiological and molecular mechanisms involving the ENS are responsible for the control of the energy balance, and for the nutritional therapies used in patients with obesity, type 2 diabetes mellitus, inflammatory bowel diseases (IBDs), bariatric surgery and cancer-associated anorexia [5-9].

In the 1950s, the chemist Linus Pauling established a relationship between decreased longevity and obesity [10]. At this time, with the advent of studies involving the mechanisms that modulate appetite control, it was recognised that the hypothalamus is the "appetite centre". In rats, some researchers observed that lesions in the lateral hypothalamus produced anorexia (hunger centre) and lesions in the ventromedial nuclei of the hypothalamus produced obesity (satiety centre) [11-14].

More recently, the discovery of cloned leptin in 1994, which is produced and secreted by adipose tissue, provided some evidence that appetite control could also be modulated by peripheral tissues [15].

**Figure 1:**
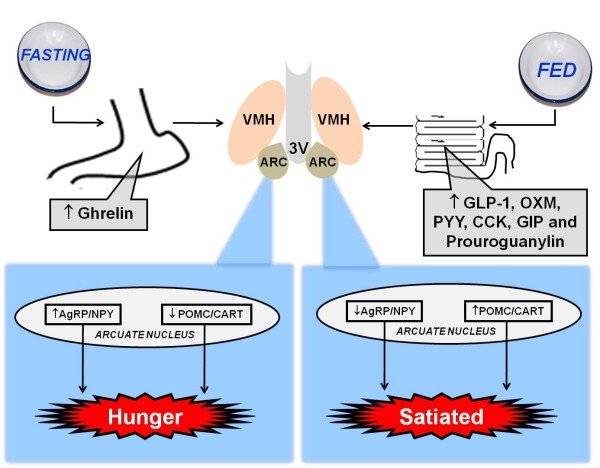
**Role of gut hormones in fasting (right) and postprandial (left) state**. During fasting, occurs greater release of ghrelin from the stomach that acts in arcuate nucleus via vagus nerve stimulating hunger. During the fed state (postprandial), occurs release of anorexigenic hormones (GLP-1, OXM, PYY, CCK and GIP) that reach the arcuate nucleus via the brainstem and vagus nerve, activating the satiety. GLP-1: glucagon-like peptide 1, OXM: oxyntomodulin, PYY: peptide YY, CCK: cholecystokinin, GIP: glucose-dependent insulinotropic polypeptide.

In relation to the mechanisms of intestinal hormonal action, the beginning of the food intake process results in the release of anorexigenic hormones, such as peptide YY (PYY), the glucagon-like peptide 1 (GLP-1), oxyntomodulin (OXM), the glucose-dependent insulinotropic polypeptide (GIP), cholecystokinin (CCK) and prouroguanylin (Figure 1). Likewise, the CNS receives and integrates several factors, adjusting the energy balance in accordance with energetic necessity. Overall, the secretion of anorexigenic hormones (including PYY, GLP-1, OXM, GIP, CCK and prouroguanylin) and the activation of neuropeptides, such as POMC and CART, occurs in the postprandial state. On the other hand, the greater release of the orexigenic hormone ghrelin and the activation of the neuropeptides AgRP and NPY occurs in the fasting state [16-18] (Figure 1). In the next step, the main gut hormones that influence energy homeostasis are summarised (Table 1).

**Table 1:** Summary of the main gut hormones that influence the energy homeostasis

Gut hormones (receptor)	Secretion site	Action
PYY (G protein-coupled receptors)	L cells of gut	↓ food intake and delays gastric emptying
GLP-1 (GLP-1R)	L cells of gut	↓ food intake, releases insulin, hand out as incretin, ↓ glucose levels and delays gastric emptying
OXM (GLP-1R)	L cells of gut	↓ food intake
CCK (CCK1 and CCK2)	I cell of small intestine	↓ food intake
Uroguanylin and Guanylin (GUCY2CR)	Intestinal epithelial cells	↓ food intake
GIP	K cells of gut	↓ food intake and glucose levels
PP (Y4 and Y5)	PP cells of pancreas	↓ food intake
Amylin (AMY 1-3)	β cells of pancreas	↓ glucose levels
Insulin (IR)	β cells of pancreas	↓ food intake and glucose levels
Glucagon (GCGR)	α cells of pancreas	↑ glucose levels and insulin secretion
Ghrelin (GHSR1)	Stomach	↑ food intake

PYY: peptide tyrosine tyrosine; GLP-1: glucagon-like peptide 1; PP: pancreatic polypeptide; OXM: oxyntomodulin; CCK: cholecystokinin; GHSR1: growth hormone secretagogue; GUCY2C receptors: guanylyl cyclase 2 C receptors; IR: insulin receptor; GIP: glucose-dependent insulinotropic peptide.

For nutrition professionals, the gut-CNS axis is considered an attractive opportunity, because foods may help to treat and prevent diseases. In this review, we discuss the fact that nutritional therapies could modify the gut flora and may reach the CNS in order to modulate the food intake and inflammatory processes. Some nutritional therapies that are known to modulate the gut-CNS axis via physiological and molecular mechanisms are also discussed.

### The main underlying mechanisms behind the connection between microbiota and the central nervous system

The components that interact to form this complex brain-gut communication is bidirectional, with stimuli from gastrointestinal tract (GIT) that influences the brain functions and messages from the brain that may alter some GIT activities, such as motor, sensory and secretory [19]. It was demonstrated that this link occurs via the vagus nerve to the brainstem, and via spinal afferents to the spinal cord [20]. Recently, Bravo et al. [21] showed that vagotomized mice did not exhibit behavioral and neurochemical effects that *L.rhamnosus *exerts in CNS, evidencing the correlation of the vagus nerve in the direct communication between the bacteria and the brain.

Moreover, the serotonin (5-HT) levels and hypothalamic-pituitary-adrenal (HPA) axis may also participate in this connection. All connections are involved with modulation of infections and inflammatory diseases, such as obesity, type 2 diabetes mellitus, ulcerative colitis, Crohn's disease, as well as with behavioural problems and psychiatric disorders, such as cognition, mood, emotion, stress and anxiety [21,22].

### Diets and microbiota: A general overview

The microbes that reside in the gut favors the harvest of energy from food, influence the metabolic profiling of organs and exerts nutritional and protective effects on the intestinal epithelium and immune system [23-25]. Moreover, the microbiota consists mainly of bacteria that are divided in two main phylotypes: *Bacteroidetes *and *Firmicutes *[26,27].

Supporting normal digestion and host metabolism, gut microbiota is able to expand nutrient availability, releasing energy through fermentation of otherwise non-digestible oligosaccharides or by modulating absorption. The short chain fatty acids (SCFA), which are the major metabolic products of anaerobic bacteria fermentation, are an important energy source for humans, being used by colonocytes, liver and muscle. It has been reported that 5 to 10% of human basal energy requirements are provided by SCFA [28-31].

Since the interactions of microbes with host leads to a complex balance of host genes, alteration of microbiota population can cause several metabolic disorders.

**Figure 2:**
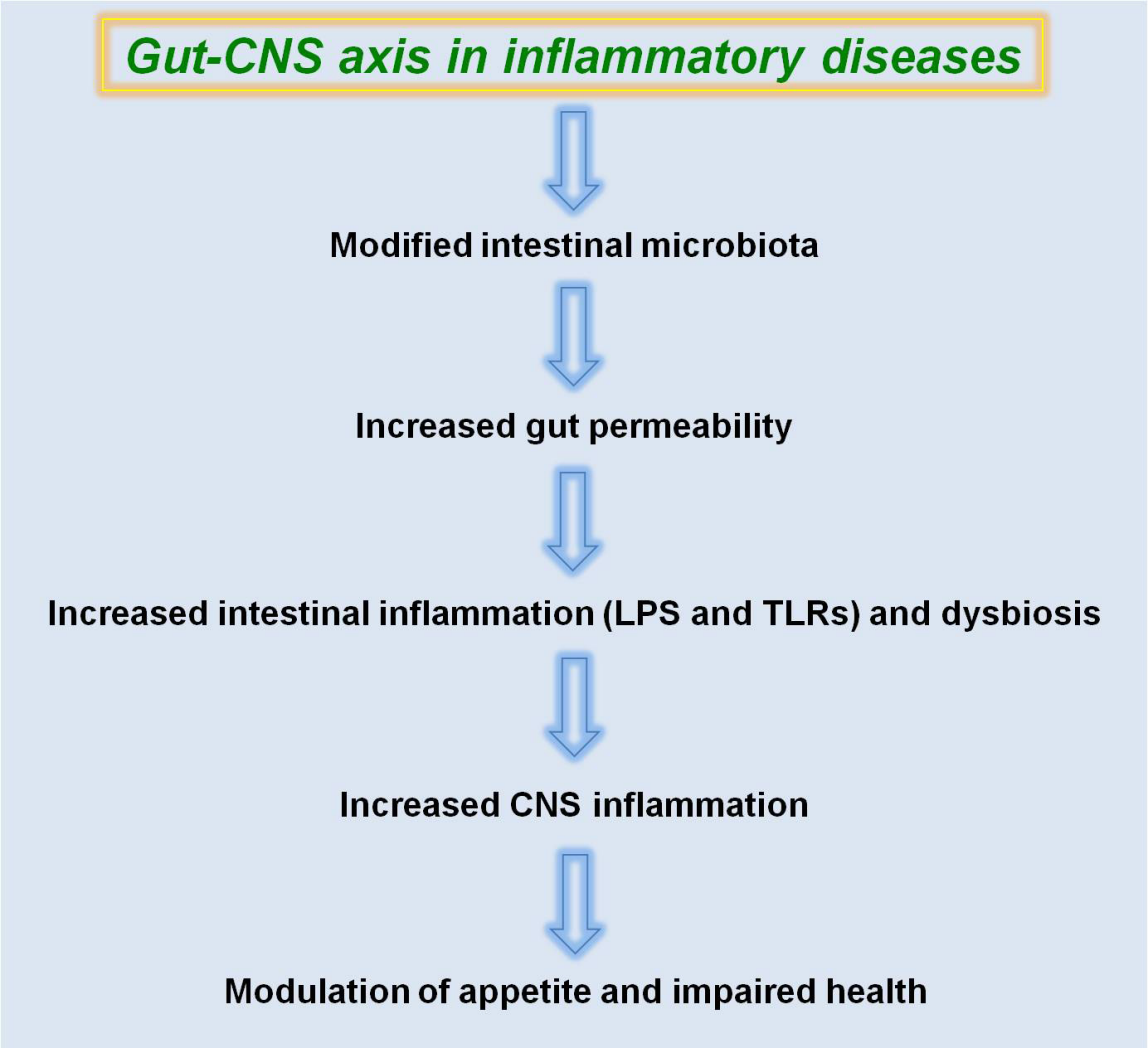
**Role of gut-central nervous system axis on inflammatory diseases**.

Recently, Cryan & O'Mahony [19] suggested that numerous conditions may modify the microbiota, such as obesity, IBDs, antibiotics, infections and diets [22,32]. Therefore, the roles of the gut-CNS axis on metabolic diseases, focusing on the physiological and nutritional aspects are summarised in the Figure 2.

### Obesity and type 2 diabetes mellitus

Since it is known that the microbiota is related to energy homeostasis, digestion of nutrients and metabolism, some low-grade inflammation-related diseases have emerged as an attractive opportunity for researchers.

The first studies to observe that obese microbiota have an enhanced ability to absorb energy from the diet were described by Jeffrey Gordon's group [33,34].

Cani et al. [35] proposed that both obesity and type 2 diabetes mellitus can be characterised by increased lipopolysaccharide (LPS) levels. For instance, in the presence of diet induced obesity (DIO), the LPS concentrations are higher than in the fasting state. In addition, either DIO animals or those submitted to subcutaneous injections of LPS represent enhanced LPS-containing microbiota, as well as glucose and body weight gain. Likewise, these authors were the first to demonstrate that "metabolic endotoxaemia" initiates obesity [35].

Recently, it has been shown that food rich in saturated or trans-fatty acids stimulates inflammatory markers [9,36-40]. Raybould [41] suggests that intestinal inflammation is associated with obesity due to high LPS levels. In 2011, it was shown that the ingestion of trans fatty acids during gestation and lactation led to an increase in blood LPS levels, the activation of inflammatory signalling in the hypothalamus and an increased food intake in adult offspring rats [9]. Moreover, the same studies [27,42] observed the presence of intestinal inflammation in different models of obesity, such as eating a high-fat diet, rich in saturated fatty acids and genetic obesity.

When evaluating inflammatory markers in CONV mice fed with a high-fat diet, Ding et al. [43] observed increased body weights and activation of gut TNF-α mRNA expression. Likewise, Caricilli et al. [27] showed increased blood LPS levels in toll-like receptor 2 (TLR2)-deficient mice when compared to wild-type mice. TLR2 deficient mice showed activated phosphorylation of janus kinase (JNK), TLR4 and phosphorylation in serine of the insulin receptor substrate-1 of several tissues [27]. In this work, the authors suggest that an increase in LPS levels together with TLR4, in the absence of changes of TLR2, result in a compensatory action that may lead to increased activation of TLR4. Together, this would contribute to insulin resistance in TLR2-deficient mice [27]. Likewise, in a previous study [9] performed with adult offspring rats from mothers fed trans fatty acids during gestation and lactation, increased blood LPS levels and hypothalamic TLR4 expression were seen with no change to hypothalamic TLR2 expression. Moreover, the increase of blood LPS provoked by a high-fat diet has also been shown by other recent studies [44,45]. It has also been shown that the actions of fatty acids are aggravated by physiological ligands of G-protein-coupled receptors, such as GPR40, 41, 43, 84 and 119, and, therefore, it may be involved in the progression of several inflammatory diseases [46].

Another TLR described to influence microbiota is TLR5. Vijay-Kumar et al. [47] demonstrated that mice deficient in TLR5 exhibit obesity, hyperphagia, dyslipidaemia, hypertension and insulin resistance, and that they also show an altered composition of gut microbiota, such as increased *Firmicutes *(54%) and lower *Bacteroidetes *(39.8%).

In addition to inflammatory processes, non-alcoholic fatty liver disease (NAFLD) is a typical hepatic manifestation that has been found to be obesity-related. Recently, inflammasome-deficient mice were shown to have modifications of gut microbes population through the influx of TLR4 and TLR9 agonists into the portal circulation. Therefore, increased hepatic inflammation levels can lead to the development of NAFLD and obesity [48].

An important study that demonstrated the relationship between intestinal bacterial and obesity in humans was published by Wu et al. in 2011 [49]. In this study, it was found that an increased fat intake and low dietary fibre are associated with the modulation of intestinal microbiota. The authors of this study showed that animal proteins and saturated fatty acids are associated with increased *Bacteroidetes *levels, and that diets containing carbohydrates but lacking meat and dairy products increased *Prevotella *levels. Together, these facts create a profile of weight gain and gut inflammation-related bacteria.

DIO experimental models also indicate low expression of tight junction proteins in the gut, and the increasing in intestinal permeability [41]. Brun et al. [50] found in either *ob/ob *and *db/db *mice, an alteration in the intestinal permeability same when mice were submitted to standard chow consumption. In rats fed with hyper-lipidic diet also was observed an increase of intestinal permeability through the reduction of tight junction proteins, such as claudin 1, claudin 3 and junctional adhesion molecule-1 [51].

Collectively, several obesity models have observed that a major determinant of intestinal permeability is the intercellular tight junction proteins. Tight junctions are organised by the same transmembrane proteins, such as occludin, claudin and junctional adhesion molecule-1 [52-54]. Therefore, these transmembrane proteins interact with Zonula Occludens (ZO-1-3), which anchors the transmembrane proteins [55] provoking an increase of intestinal permeability. The increased intestinal permeability is thought to be associated with a higher activity of pathogenic bacteria and inflammatory processes. In summary, some studies have described that the main tight junction proteins responsible for this intestinal permeability are ZO-1, myosin light chain, occludin, claudin and junctional adhesion molecule 1 [44,50,52-54,56,57].

Several papers suggest that saturated fatty acids might enhance the blood LPS levels through GPRs, possibly secreted by gut cells, may affect the CNS and alter numerous central inflammatory markers. In addition, the increased intestinal permeability aggravated by a high-fat diet and LPS may also be responsible for altered epithelial barrier function, and it is therefore possible that the high prevalence of obesity and type 2 diabetes mellitus is connected with an altered gut microbiota-CNS axis.

### Bariatric surgery

In the middle of the 1950s, Kremen et al. [58] postulated that bypass surgery in dogs reduced food absorption. Recently, in humans, bariatric surgery has been found to be a procedure which results in patients rapidly losing weight, accompanied by the resolution of type 2 diabetes mellitus and a reduction of cardiovascular deaths [45,59]. However, the mechanisms underlying the improvement of metabolic parameters have not been fully elucidated. Likewise, Evans et al. [60] have shown that obese patients had an increase of blood PYY levels and GLP-1 was restored to normality after gastric bypass surgery compared to patients with normal weights. Short-term Rouxen-Y gastric bypass (six months) was able to activate PYY and GLP-1 secretion, and stimulated the satiety in response to a liquid-meal intake in normal, glucose-tolerant obese patients [61]. Falkén et al. [62] reported that patients had a progressive rise of the GLP-1 and OXM concentrations after gastric bypass and that this procedure favours weight loss and improved insulin sensitivity.

**Figure 3:**
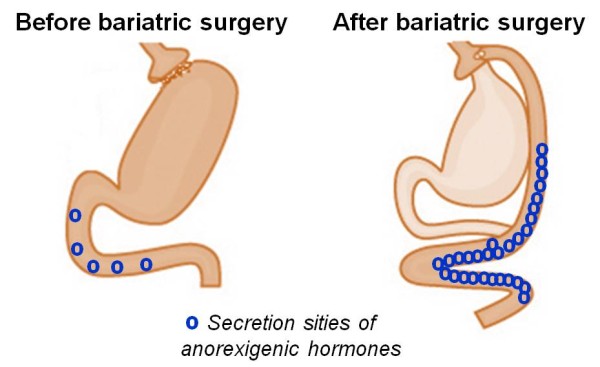
**Secretion sities of anorexigenic hormones, such as glucagon-like peptide 1 (GLP-1) and peptide YY (PYY) are increased after bariatric surgery**.

Collectively, the published data has shown that, after bariatric surgery, numerous gut hormones can reduce the appetite and normalise glucose homeostasis, and that the main actions are modulated through GLP-1, PYY, OXM, ghrelin, insulin and leptin [63]. Moreover, an increased secretion of anorexigenic hormones, such as GLP-1 and PYY occurs with bariatric surgery (Figure 3). It is also possible to speculate that the bariatric surgery might modulate the intestinal permeability and enhancement of anorexigenic gut hormone secretion, alongside the reduction of inflammation seen in obese patients, but this has not been evaluated. Collectively, it is known that theses anorexigenic hormones might activate POMC and CART neuropeptides in order to reduce the food intake and body weight, and also help to moderate energy expenditure.

### Inflammatory bowel diseases (IBDs)

The IBDs, that affect the health of humans, include ulcerative colitis, Crohn's disease and irritable bowel syndrome (IBS) [64-66]. Macfarlane et al. [67] have suggested that the appearance of these diseases may be due to modified gut microbiota, or as a consequence of local inflammation. However, a recent study has shown that the intestinal wall of either inflamed or non-inflamed guts [68] may be associated with severe disease. Likewise, human studies of patients diagnosed with IBDs have observed increased TLR2, TLR4 and TLR5 expression in the gut wall [69,70], and other studies have reported an increase of the IL-6, IL-8, TNF-α and interferon-gamma levels [71-73]. Therefore, it is possible to observe that in IBDs that have an inflammatory status higher than that of obesity, more severe disease symptoms are seen. In order to investigate the effects of neuro-inflammation in animals submitted to experimental IBD, Wang K, et al. [74] observed increased IL-6 mRNA expression in both the colon and brain of these animals when compared to control animals.

Collectively, it can be speculated that higher levels of gut-related inflammation lead to a reduction of food intake and malnutrition due to the activation of cytokines in the CNS. According to Pavlov & Tracey [75], the autonomic nervous system plays a key role in the control of the brain in moderating the immune system and inflammation.

In summary, several studies have reported that inflammatory bowel problems are linked to a reduction of *Lactobacillus spp *and *Bacteroidetes*, and an increase of the *Firmicutes-Bacteroidetes *ratio [76-79]. These changes in intestinal flora are aggravated through alterations in the immune system that underlie disease pathogenesis.

### Nutritional therapies that improve metabolic diseases through the gut-CNS axis

While microbes have been used to study the underlying mechanisms of inflammatory diseases and insulin resistance, numerous researchers have also stated that nutritional components could be used as a strategy to combat the gravity of these abnormalities. Historically, the Greek physician Hippocrates, "The father of medicine", reportedly said "Let your food be your medicine, and your medicine be your food".

Among the nutritional components that support a healthy intestinal microbiota, we highlight the dietary fibres, probiotics and prebiotics [80-84]. The improvements include a reduction of systemic and local inflammation, as well as less intestinal pain and discomfort, when both probiotics and prebiotics are used [80,85,86]. In addition, other studies have shown an inhibition of bacterial translocation and a reduction of intestinal permeability with the use of these nutrients [87,88].

One study demonstrated that the use of oligofructose, a prebiotic, enhanced the levels of *Bifidobacterium spp*., and improved insulin sensitivity, as well as restoring inflammatory status through decreased endotoxaemia metabolism [80].

Oligofructose and resistant starches have been demonstrated to increase short-chain fatty acid-induced GLP-1 expression [89], and to reduce ghrelin expression [90]. Moreover, in a recent review the nutrients and diets that stimulate the anorexigenic gut peptides, reduce the food intake and break off the obesity were summarised. The main nutrients and diets that can increase the GLP-1, CCK, GIP, OXM and PYY secretion are dietary fibre, dairy products, unsaturated fatty acids and a normal calorie diet [2].

Recently, it was observed that a prebiotic-enriched diet reduced *Firmicutes *and increased *Bacteroidetes *levels, as well as improving glucose sensitivity, body fat, inflammation and oxidative stress [91]. Furthermore, a recent review suggested that SCFA providers provided of dietary fibers, such as propionate and butyrate, induce the reduction of food intake by increasing leptin secretion and reducing pro-inflammatory cytokine expression [92]. These dietary fibres are fermented by microbiota, and higher butyric acid levels were found in animals fed with diets containing a mixture of oligofructose and raffinose [93].

Necrotising pancreatitis patients with diarrhoea received semi-elemental nutrients via jejunal feeding, and an increase in faecal short-chain fatty acids, such as acetate, propionate and butyrate were found when compared to pre-treatment levels. In addition, a resolution of diarrhoea episodes was seen in approximately 66% of patients. Therefore, this study suggests that dietary fibre supplementation is an excellent method for the improvement of healthy intestinal microbiota, and results in reduced symptoms of dysbiosis [81].

Concomitant to numerous "healthy" nutrients, it is possible that unsaturated fatty acids, such as omega-3, are an attractive option for the improvement of inflammatory processes, and that this can be modulated by the physiological ligand of these fatty acids, GPR120 [94,95].

In relation to high-grade inflammatory diseases, such as a provoked by Human Immunodeficiency Virus type1 (HIV-1), patients were shown to have infections in lymphoid tissue, alterations of intestinal microbiota and impaired symptoms of Acquired Immunodeficiency Syndrome (AIDS). Therefore, the use of probiotic diets is suggested for the prevention of progression of HIV-linked infections [96].

It has been recognised that obese, insulin-resistant and IBD subjects represent a group requiring moderation of intestinal microbiota due to a higher risk of the development of cancer. This is because the mechanisms by which intestinal bacteria induce carcinogenesis are thought to be via chronic inflammation, immune system evasion and immunosuppression. Conversely, the probiotics used have also emerged as an possible mechanism for the reduction of the pro-inflammatory status seen in cancer patients [97].

**Figure 4:**
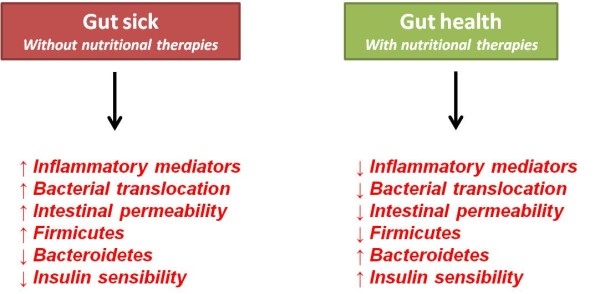
**Summary of main physiological and metabolic alterations that modify the gut sick and health before and after the nutritional therapies**.

Collectively, this topic has summarised the main physiological and metabolic alterations that modify illnesses of the gut both before and after nutritional therapy (Figure 4).

Although nutritional compounds are important for the improvement of health, changes to diets, such as an increase of either fruit or vegetable consumption, as well as a reduction of refined carbohydrates and saturated and trans-fatty acids [98,99], are required, as food restriction can prevent obesity in humans [98,99] and mice deficient in the TLR5 [47]. Furthermore, micronutrients and macronutrients from existing diets are targets for gut health and strengthening of the immune system [100].

**Figure 5:**
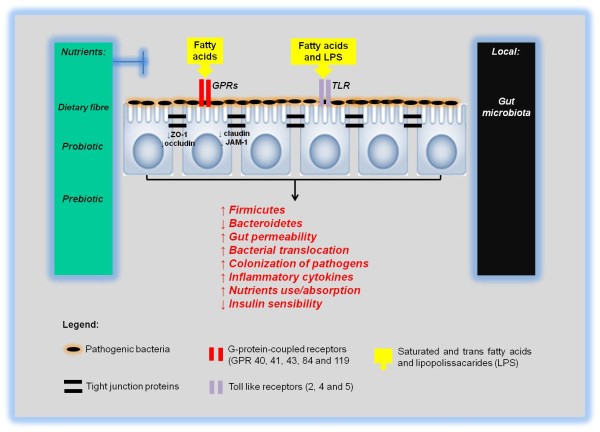
**Role of intestinal microbiota on development or prevention of inflammatory diseases via dietary fibers, probiotic or prebiotic in the peripheral tissues and central nervous system**. ZO-1: Zonula Occludens, JAM-1: juntional adhesion molecule 1.

The studies discussed in this review collectively support the emerging view that microbiota contribute to metabolic disease, and suggest that an impaired diet quality may promote the development of inflammatory diseases. However, some nutrients that have been studied, such as dietary fibres, and probiotic and prebiotic nutrients, along with bariatric surgery, are one possible option for the maintenance of intestinal health, and the improvement of the gut-CNS axis (Figure 5).

In summary, the discoveries in understanding these foods and nutrients could help to regulate the gut-CNS axis, but remain a challenge for nutritionists and scientific investigators. Therefore, future research must be focused on looking to improve the effectiveness of diets for the prevention of inflammation between the gut-CNS axis, as well as for the maintenance of microbial homeostasis of the gut.

## Competing interests

The authors declare that they have no competing interests.

## Authors' contributions

GDP performed the design of the study, researched and discussed the articles and written the paper. TOM, FP, JCR, RVTS and FSL also researched and discussed the papers. All authors read and approved the final version manuscript.
